# The American Dental Society of Europe

**Published:** 1901-12

**Authors:** 


### THE AMERICAN DENTAL SOCIETY OF EUROPE.

Upon another page of this number will be found an earnest appeal to the members of the National Dental Association to change the date of meeting at Niagara next year from August to July. This is desired to accommodate those members of this Association who may propose to attend the annual meeting of the American Dental Society of Europe, to be held at Stockholm in August next.

The readers of this journal are aware that a change from August to an earlier period has been repeatedly advocated. The heated term has always been a detriment to this meeting, and it remains a mystery why the majority persist in making use of August as the meeting month of the year.

If May or early June were selected it would give an opportunity for those who desire to attend the meetings usually held in Europe in August. The climate there is altogether different at this period from that in America. Whether it would be wise to change it this year to accommodate the American Dental Society of Europe remains for the Executive Committee to determine, but it is hoped that the subject of a permanent change may officially be brought up next year either by this committee or the President in his annual address.

